# Attachment-Based Mentalization Profiles of Iranian Children: A Mixed-Method Approach

**DOI:** 10.3390/children11020258

**Published:** 2024-02-17

**Authors:** Masoumeh Zandpour, Majse Lind, Carla Sharp, Jafar Hasani, Farzin Bagheri Sheykhangafshe, Jessica L. Borelli

**Affiliations:** 1 Department of Psychology, Faculty of Humanities, Tarbiat Modares University, Tehran 14117-13116, Iran; farzinbagheri@modares.ac.ir; 2 Department of Communication and Psychology, Aalborg University, 9000 Aalborg, Denmark; mlind@ikp.aau.dk; 3 Department of Psychology, University of Houston, Houston, TX 77024, USA; csharp2@central.uh.edu; 4 Department of Clinical Psychology, Faculty of Psychology and Educational Sciences, Kharazmi University, Tehran 15719-14911, Iran; hasanimehr57@khu.ac.ir; 5 Department of Psychological Science, University of California, Irvine, CA 92697, USA; jessica.borelli@uci.edu

**Keywords:** reflective functioning, mentalizing profiles, Iranian children, cross-cultural research, qualitative analysis

## Abstract

Mentalization, operationalized as reflective functioning (RF), is the ability to understand one’s own and another’s mental world implicitly or explicitly. RF is a newly discovered research field in Iran and is largely under-studied in Eastern cultures in general, underscoring the high need for cross-cultural studies in this field of research. A qualitative method was used to examine the ability to understand, process, and respond to high-arousal attachment situations in typical and clinical populations of Iranian children recruited from a Tehran primary school. A well-known semi-structured interview commonly used to assess RF in children was used to collect data. Required information on internalizing and externalizing symptoms, demographic information, and all formal diagnoses of children were collected by parents. The results indicated the identification of four different profiles of RF in children, one of which was adaptive, while the other three were maladaptive. Also, the results showed that typically developing children and those having a high social and economic status (SES) were characterized as having a more adaptive profile of RF, while children from the clinical population and those with a low SES reported a more maladaptive profile (passive mentalizing, helpless mentalizing, narcissistic mentalizing) of RF. The present study is an important step in increasing our understanding of the development of mentalization in children and has significant educational and clinical implications.

## 1. Introduction

Mentalizing, often operationalized as reflective functioning (RF), refers to the mental ability to reflect on and interpret one’s own and other people’s behaviors in terms of internal mental states such as feelings, thoughts, intentions, and desires [1]. As such, mentalizing is a fundamental capacity necessary to make sense of oneself and navigate successfully in the social world [2]. Children’s mentalization develops gradually from early childhood and relies heavily on the quality of early attachment figures, with parents being the typical main figures. That is, adequate mentalization is best established in an environment where parents treat their child as a psychological being with a mind they curiously and consistently try to understand, mirror, and interpret [3]. Thus, the child’s mentalizing mind grows out of other mentalizing minds. Developing adaptive mentalization in childhood is crucial because it serves as an underlying ability to understand, process, and respond appropriately to attachment-aroused situations and has been associated with a coherent sense of self [4] and resilience [5], whereas compromised mentalizing has been linked with greater psychopathology [6,7].

A growing body of research has begun to study mentalizing within specific cultures within the last few decades [8], such as researchers Bernal and Sáez-Santiago, 2006; Roland, 1996; Kirmayer, 1987; Ryder et al., 2018; and Aival-Naveh et al., 2019 [2,8,9,10,11]. Aival-Naveh and colleagues [2] reviewed cross-cultural research on mentalization, aiming to identify potential culturally dependent mentalizing profiles, potential mediators, and whether the link between mentalizing and psychopathology remained across cultures [2]. Based on a total of 112 papers from more than 45 countries including both child and adult populations, the authors showed that individuals from collectivistic cultures tended to be more other-oriented and score higher on emotional reactivity, whereas individuals from individualistic countries were more self-oriented, indicating cultural-dependent mentalizing profiles. However, these differences were most evident in the more verbal, explicit, and slow mentalizing tasks and less distinct in the nonverbal, implicit, and fast assessments, insinuating that the differences are less clear and may be due to language barriers. Value preferences and parental characteristics were identified as mediators. These authors’ cross-cultural research studying the link between compromised mentalizing and psychopathology was sparse and predominantly based on self-reports. However, other existing research indicates that mentalizing is also disturbed in the context of psychopathology within collectivistic countries [2]. In Iran, considering children’s opposition to parents as disrespectful, prioritizing the needs and desires of others instead of one’s own needs and desires, and suppressing emotions are among most important factors influencing why Iranian children are lagging behind their Western peers in understanding the mind. Parenting philosophies, parents’ ways of conversation [12] and biases toward understanding the experiences of others, and paying little attention to one’s point of view [13] have created certain concepts of mental states in Iranian children. Unlike children in the United States of America, Australia, and England, who are constantly encouraged to express themselves freely and participate in discussions and are exposed to a diversity of opinions during family discussions and arguments, Iranian children have less chance to be exposed to these richer and more varied experiences for developing their mental world. Although collectivist and individualist societies display differences in their social understanding and theory of mind, these changes occur in completely compatible cultural patterns [12].

Existing studies on RF in both Western and Eastern cultures have adopted a quantitative approach to understanding this construct. Quantitative methods are useful for creating generalizations about groups, but oftentimes it can also be helpful to supplement quantitative approaches by studying a phenomenon with qualitative analyses. By examining our data and narratives at the level of individual responses, qualitative data analytic methods can enrich our understanding of the cognitive and emotional processes inherent in psychological phenomena, particularly when examining under-studied topics within under-studied populations. Qualitative analyses can then complement what has been revealed from quantitative approaches, in that they typically reveal more fine-grained conclusions regarding psychological processes. Thus, the current study adopts a phenomenologically rich, first-person account of the child’s mental world [14], ideal for identifying culturally sensitive mentalizing profiles. With this qualitative study, we contribute to the literature by filling a gap within the existing cross-cultural literature [2] in expanding our knowledge base of the variability in mentalizing patterns in Iranian children. To accomplish this goal, we recruited both typically developing children and non-typically developing children suffering from various psychopathologies from the collectivistic country of Iran. The inclusion of both typically developing and clinical children should provide us with sufficient variability for different mentalizing profiles to manifest. With their parents’ consent, the children engaged in a semi-structured Child Attachment Interview [15] that was recorded, transcribed, and content-coded for potential attachment-based mentalizing profiles.

Qualitatively, given the desire to not impose any Western-based mentalizing classification on the Iranian children’s narratives, the type of mentalizing profiles were identified inductively. Quantitatively, however, guided by extant research in North America and Western Europe, we expected to find more adaptive mentalizing profiles among the typically developing children and more compromised mentalizing profiles among the clinical population.

We also examined the role of SES concerning the mentalizing profiles. SES is one of the important moderators for self-regulation and emotion regulation [16], and the way people process and navigate in the social world is fundamentally and neurocognitively dependent on it [17]. Living in a low-SES context is associated with family instability and higher cortisol levels, and has a significant negative impact on the development of language abilities, cognitive functions, executive functioning, and theory of mind [18]. SES is one of the important factors that affect children’s social understanding and the speed of theory of mind development. According to their level of SES, people internalize and display different levels of individualistic or collectivist values [19]. In low-SES families, parents report lower RF and children experience more stress in parent–child relationships. They are usually deprived of a rich language environment to solve problems in the family. Also, in terms of the number of words, the variety of words used, and the complexity of sentences, there are significant quantitative and qualitative differences in families with a low SES compared to those with a high SES [20]. Thus, we expected an association between having a lower SES and exhibiting more maladaptive mentalizing.

## 2. Methods

### 2.1. Participants

A total of 153 Iranian children aged 8 to 11 years (*M*_age_ = 9.41, *SD*_age_ = 1.10, *M*_male_ = 45.8, *M*_female_ = 54.2, *SD*_gender_ = 0.499) were recruited for this study. The children were either typically developing children (*N* = 122) recruited from primary schools or recruited from a psychological clinic in Tehran (*N* = 31) from November 2020 to January 2022. The group of typically developing children included 83 female and 70 male children, with the majority (*n* = 122, 54 male, 68 female; 80%) of children having no formal diagnoses or history of psychological treatment (i.e., based on the parental report). The children from the clinic (*n* = 31, 16 males, 15 females) were formally diagnosed by the psychology clinic in which they were also being recruited; of these, most reported anxiety and/or depression (*n* = 27), followed by attention deficit hyperactivity disorder (*n* = 3), and oppositional defiant disorder (*n* = 1). The participants came from different socio-economic backgrounds. They were recruited from both rich and poor areas of Tehran based on their parents’ education, parents’ jobs, place of residence, and the family income reported by parents to ensure maximum diversity among the participants (43.7% low SES and 50.3% high SES). A low-SES family, according to the parental report, was defined as a family with a low income, low-level jobs, low level of education (mostly primary or below diploma), and living in a poor or marginalized area. High-SES families were families with high-level jobs and high incomes; most of them had university education and lived in good neighborhoods in the city.

### 2.2. Assessment

To screen typically developing children with respect to mental illness, the mother version of the CBCL (CBCL/6-18; [21]) was applied. This questionnaire assesses the emotional–behavioral problems as well as the academic and social abilities and competencies of children aged 6 to 18 from the parent’s point of view. The school age checklist contains 118 problem behavior questions. This form measures the following: Child adaptive behaviors (113 items), which are divided into three scales: Activities, Social Competence, and School Competence, which are each scored on a 3-point scale (0 = Not True, 1 = Somewhat or Sometimes True, 2 = Very True or Often True). Problem behaviors (20 items), which are divided into two broadband scales (Internalizing and Externalizing) and eight narrowband scales that assess emotional behavioral problems or syndromes, including Aggressive Behavior, Anxiety/Depression, Attention Problems, Rule Breaking, Somatic Complaints, Social Problems, Thought Problems, and Withdrawal/Depression. Also, Total Competence and Total Behavior Problems scores were measured.

This measure has previously been validated and used in samples with Persian youth [22]. The internal consistency of this scale in the Iranian sample is relatively high and its alpha range was 0.85 [22]. The Cronbach’s alpha coefficient was 0.69 for the Internalizing scale and 0.88 for the Externalizing scale.

The data of this study were collected using the full-length Child Attachment Interview [15]. The CAI is a well-known and effective instrument for measuring mentalization in children between the ages of 7 and 13. The CAI is a semi-structured interview with 15 items. Items are designed to activate attachment schemas in children by asking RF-related questions, which offers the opportunity to assess how well children can mentalize around attachment themes (e.g., the child is asked to provide three words to describe themselves/what it is like to be with dad/mom, as well as being asked what happens when mom/dad becomes upset with them, if anyone close to them has ever died, and if they ever feel that their parents don’t like them). These questions measure issues such as parental conflict and fear and loss, which are examples of challenging situations within the CAI. It is assumed that children’s reactions to difficult situations are aimed at coping with/controlling these situations. This means that children try to respond to them according to their cognitive and emotional abilities, to reduce the suffering caused by a difficult situation for themselves and make the situation bearable. They process experiences in different ways that could be adaptive or maladaptive.

### 2.3. Procedure

Ethics approval was obtained through the ethics committee of Kharazmi University. Participants who expressed interest in the study contacted the first author of this paper, who then did an initial screening to determine eligibility. The first author was introduced to a psychology clinic through the university specializing in emotional and behavioral disorders in children and adolescents. Also, through the Education Department of Tehran, the main researcher was introduced to several primary schools in different areas of the city. To recruit typically developing children, after obtaining the necessary permits, a demographic questionnaire and the CBCL/6-18 [21] were sent electronically by the child’s school or community clinic to mothers who were potentially interested in having their child participate in the research. The CBCL was included as a self-report screening tool to ensure that the typically developing children did not suffer from mental disorders according to their mothers. Due to COVID-19, parents were able to choose the format of the CAI interview, with the resultant sample including *n* = 95 interviews conducted over the phone, *n* = 54 in person, and *n* = 4 through video conference. The in-person CAI interviews were completed in a private room at the community clinic or a private location at the child’s school while their parents were in a room nearby. All parents gave written consent to have their children participate in the research study. They could decide to withdraw from the study at any time and the interview would end if the child did not want to be interviewed. The interview took approximately 38 min to one hour. A pilot study with eight interviews was first conducted and mentored by a senior researcher. The purpose of this pilot study was to familiarize ourselves with the interview and to make any necessary adjustments before the large data collection could begin. All interviews were conducted by the first author in the span of February 2021 to October 2021.

### 2.4. Coding

Thematic analysis with an emphasis on reflexivity coding was used to analyze the data. This method emphasizes the insight and depth of the researcher’s interpretation, and the process of analysis and coding using a single coder can also lead to good and valid coding [23]. The thematic analysis provides a robust, systematic, six-phase framework for extracting themes in which the researcher immerses himself or herself in the data to identify patterns in the dataset [23,24,25,26]. The coding procedure can be conducted either as a small group or with a single coder [25]. To ensure reliability of the analysis, the coding was completed by the first author in collaboration with a co-rater. The interviews were conducted in official Farsi, the language the children spoke at school. The coders were all Farsi speakers and natives of Iran.

Step 1: The process of writing down the children’s narratives makes the first author familiar with and immersed in the data. This is the first and most important stage of thematic analysis because it helps the researcher to be aware of the depth and richness of the content through the goals and questions of the research [26]. For this purpose, qualitative data was implemented and written word for word. Then, to create a general sense of the data and also to further immerse the researcher in the data, the researcher listened to the audio recording again, read the executed text several times, and compared it with the verbatim transcript to fix possible errors.

Step 2: In the Step 1 data collection, 1350 meaning units or verbatim phrases were extracted, which were finally classified into 150 initial categories. The answers were categorized very carefully and continued to be reviewed and revised, and then the two raters simultaneously began analyzing them verbatim to find their basic concepts or initial codes. In the process, several sessions were held and the resulting codes were reviewed. The codes that had the most convergence were maintained and the rest were removed; that is, codes that received 50 to 100 percent absolute agreement were retained and the rest were removed.

Step 3: The two raters independently began the analysis with a long list of initial codes and attempted to identify relationships between and across the dataset to identify sub-themes [27]. Next, the first author analyzed the qualitative data to find main themes as well as master themes. According to the objectives of the research, i.e., the study of mentalizing profiles in children’s social cognition, the researcher needed to go beyond explicit meaning to capture implicit meaning, and perform more in-depth analysis. This helped to find latent meanings that were not explicitly stated [23]. This is a recursive process, not a linear one, and the researcher must constantly review the steps and consider new topics and themes [28].

Step 4: The first author corrected all of the categorized themes and classified them more coherently and systematically.

Step 5: The themes were defined, refined, and organized into a coherent format. 

Step 6: At this stage, a brief, coherent, and logical report of the analysis process and the resulting themes was written in a way that could convince the readers of the merit and validity of the analysis [23].

After finishing the analysis, the complete report of what had been carried out along with the results was sent to another rater as well as two other professors (a methodologist and a clinical psychologist specializing in psychoanalysis, who both had supervised RF-related projects in the past and were fully familiar with RF concepts). At the same time, the findings were reviewed by all three raters. Then, in a joint meeting with all four raters, a conclusion was made. All major themes and sub-themes reached 100% absolute agreement by all four raters, but some themes were renamed and replaced with more appropriate names.

After identifying the master themes, the first author and another coder (a child therapist) began another coding process for the most dominating sub-themes. For this purpose, the sub-themes were carefully reviewed, and based on the most dominating sub-themes and the child’s elaborations, it was determined which profile of mentalizing each interview displayed. For this purpose, another 20 interviews, about 15% of the interviews, were sent to other raters. The kappa coefficient was then calculated to measure the inter-rater reliability between the raters. Kappa = 75%.

## 3. Results

Younger children were franker in answering the interview questions, but older children were coping more/were more in control of their speech and tried to express their words in a way that showed a more favorable image of themselves and protected their self-esteem. This could be because older children are better able to control and manage their mental states, while younger children are more transparent and predictable in expressing their mental states [29]. Also, younger children and low-SES children in all studied age groups had more verbal encouragement to answer the questions. In the interviews with the children, especially the low-SES children, the interviewer tried to ask questions indirectly to avoid harming the children, because in the pilot interview, the interviewer found that the younger children and children with a low SES showed significant distress when faced with the CAI questions. For example, when faced with the question of whether they ever felt that their parents did not love them, they would become very anxious and sometimes cry. To find out the effect of the interview on the children, the interviewer (the first author) talked with the parent, who indirectly monitored the interview process (mostly mothers) and asked him about the interview and the child’s state of mind during and after the interview. So, after having a good understanding of the interview process, the interviewer finally decided to ask questions with a negative emotional charge indirectly. For example, regarding the question that asked if some children are beaten by their parents, here the children gave their responses in three ways: either they confirmed it and said yes, or they said hurriedly and excitedly, “*No, I have never been beaten at all”*, or they would wait until the question was over and answer it. Then, the interviewer asks, “what do you think the child who is being beaten feels like?”, instead of asking, “have you ever been beaten?” Low-SES children reported the majority of parental disputes and fights and showed more despair and helplessness, while high-SES children were more likely to view parental arguments as a usual occurrence that ends well and were sure that their parents love each other. Children of a lower SES were usually less resilient to being away from their parents or losing a favorite object. For example, this narrative, related from a 9-year-old boy of a low SES: “*I can’t bear it if someone takes or breaks my toy… Imagine crying and begging parents a lot to buy you a toy, then someone comes and takes it or breaks it or loses it”*, and this narrative, related from a low-SES 8-year-old child: “*When my toy is lost, I think my parents are dead”*, are in contrast with this narrative of a 9-year-old boy with a high SES: “*When someone breaks my toy, I get upset, maybe even cry, but it just happened and I can’t do anything about it. My parents will buy me another one”*, and this narrative of an 8-year-old girl of a high SES: “*I was very upset and cried, but I forgave him because he didn’t break my toy on purpose”.* Usually, children in all age groups reported the need to be with their parents, play with their parents, spend time with their parents, and receive affection from their parents. However, in the narratives of younger children and children with low SESs in each age group, in response to the question, “what kind of dad/mom would you like to be?”, there were differences. Younger children reported more basic needs compared to older children in both groups, but older children reported psychological needs such as being understood and cared for. The narratives of older children in the low-SES group compared to the high-SES peer group had more trauma content, and they reported suppressed or frustrated needs, and the high-SES group had more exaltation and progressive content. Low-SES children in each age group reported similar needs to the younger children in the high-SES group. These children said that they like parents who buy them a toy and play with them and spend time with them. The important qualitative difference that existed in the narratives of the upper and lower SESs was that the low-SES children said that they like parents who buy them bags and shoes and buy them dolls (very basic needs), who do not fight them, do not beat them, and do not insult them. These children’s material and psychological needs were met at least, and it seemed that their efforts were more for survival than excellence. In contrast, older children with a high SES reported more high-level needs. For example, they liked parents who help them achieve their goals and desires, and who are friends, help each other, and are kind to each other, and usually, at the end of each sentence, they said that their parents are like that. Here, parent RF was an important moderator because low-SES children who reported their parents as understanding, kind, and good usually reported an adaptive profile of mentalization. According to research in the literature, in low-SES families, maternal RF is a protective factor that prevents children’s behavioral problems [20].

### 3.1. Thematic Analysis

**Figure 1:**
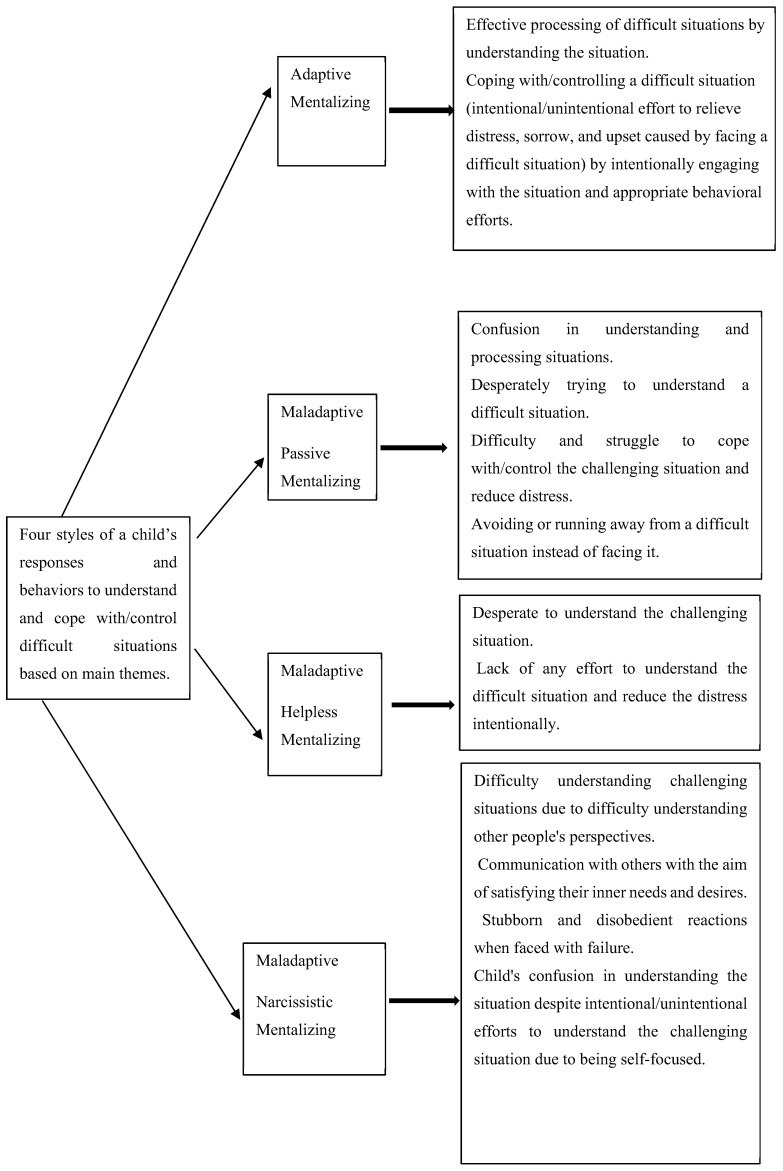
Four styles of a child’s responses and behaviors to understand and control difficult situations based on main themes.

In the process of analysis, four styles were identified through thematic analyses in which children understand, process, and cope with/control challenging situations. One of them (Adaptive Mentalizing) is efficient, and the three others, which were thereafter named Passive Mentalizing, Helpless Mentalizing, and Self-Focused Mentalizing, were deemed maladaptive and inefficient. These four styles, derived from the analysis of the main themes (see Table 1 and Figure 1 for main themes), describe how children understand, process, and respond to a difficult situation, or, in other words, children’s abilities and failures in mentalizing when describing emotions and behaviors in circumstances that typically activate their attachment schema. Each of these mentalizing profiles exhibits unique patterns that reflect the children’s vulnerability as well as their ability to mentalize situations. These profiles refer to four types of mentalization that emerged in Iranian children and explain how children perceive and process their mental world and that of others and respond to them. Finally, it should be noted that the results of this study include the identification of 4 master themes, 12 main themes, and 38 sub-themes. In Table 1, we present information about the thematic analysis.

### 3.2. Adaptive Mentalizing

An adaptive mentalizing child’s reaction to a difficult situation is a relatively mature approach based on a well-digested understanding of the situation. In this profile (86 children), the child deliberately and consciously engages with the situation (instead of withdrawing or fleeing from it) and makes a deliberate and conscious effort to find a solution to reduce their grief and suffering. The child has the desired cognitive and emotional capacity to engage with and respond to situations, and effective strategies (such as humor, engaging in pleasurable behaviors to attract the attention of others, talking to others about their discomfort, or alleviating their suffering): “*When my parents get upset with me, I get upset too, I paint for them or pick flowers from the garden… Then I kiss them and apologize”.* (It is important to note that these examples are translated. The original quotes can be shared upon request.) This form of narration was common in all age groups and both groups, but it was more frequent in 8- and 9-year-old children. Here, the child consistently tries to control the situation and reduce the damage with appropriate behavioral measures. With a good understanding of one’s inner mental world and that of others, he or she can manage communication challenges and maintain mental balance. For example, one participant said: “*When my parents are arguing, I get upset, but nothing can be done with sorrow and upset. I think, what can I do to end the argument? I bring them tea and try to say things to make them laugh so that everything goes well”.* This was very common in the age group of 10 and 11 years and in children with a high SES. These children have a good relationship with their parents and their parents can convey the concept to their child that they understand him/her. The child also had a good ability to understand the situation as well as the parents. Even if the children had experienced poverty and deprivation, this mutual understanding and good parent–child relationship seemed to offset the effects of poverty and deprivation to a large extent and did not impair the child’s mental health and RF: “*When they don’t buy me the things I want, I get upset at first, but I know they love me, but at this time, they don’t have enough money to buy what I want, so I ask them to buy it for me whenever they have money”*—an 8-year-old boy and 11-year-old girl of high SES. This type of quotation was repeated with a high frequency in the high-SES children. Children with this profile reported fewer mental health problems on the CBCL; further, they seemed more vibrant and energetic than children with other mentalizing profiles.

### 3.3. Passive Mentalizing

This profile (49 children) is identified when the child has so much difficulty understanding the situation that he or she seems confused but desperately struggles to grasp the situation. A distinctive feature of this profile of mentalizing in children is a kind of confusion and a painful struggle to understand the situation in the service of reducing pain and grief. The child has great difficulty understanding the situation and is unable to control it. The child constantly argues with himself: “*Why did this happen? Why do only I have to endure this situation? Why do only I have to be beaten? I do not know why this happened”*. This was very common in all age groups and in both groups. The strategies that the child uses to control the situation are usually ineffective because the child tries to respond to reduce the grief and suffering by withdrawing from the situation. Of course, emotional restraint to avoid unfortunate consequences and suppression of desires and inner feelings are very prominent in this profile, so the child is more prone to mental disorders such as anxiety and depression. In this profile, the child finds himself/herself unable to control the situation and endures these painful conditions with no confidence in that they can change: “*When my parents are arguing, I’m afraid. I go to my room and cry … I say, God, why are my parents fighting? … Maybe it’s my fault they are fighting? … Maybe they don’t like me anymore. … Do they not divorce?… I have no choice; I have to endure because I cannot change them or improve the situation”.* This was very common in all age groups and both groups. Due to the limited cognitive and emotional capacity of the child to adapt to a difficult situation, the child sometimes tries to make the situation bearable by denying it: “*My mother beats me a lot and doesn’t buy me anything… She makes me do hard work and insults me, but she loves me, she beats me to focus and not make mistakes”.* This type of narration was common among 8- and 9-year-old children of low SES. Although it is difficult for the child to understand his/her mental world and that of others, the child constantly struggles not to lose hope; this is a very painful endeavor. The child interprets the situation as catastrophic. In this profile, the majority of children (92%) experienced severe domestic violence, parental physical abuse, corporal punishment, psychological and behavioral abuse, and an unstable living environment that severely impaired the child’s mental health and RF capacity: “*My parents fight a lot. When my father gets angry, he wants to ruin everything and beats us very brutally…when he gets angry, I am very scared…he hits my head hard against the wall. … at that moment, I think my brain will explode”*—a 9-year-old boy of a low SES. Feelings of insecurity and a fear of losing one’s parents are omnipresent. It is worth noting that although this is seen in other profiles, this fear is more severe in passive children, and when away from their parents, they show more and more anxiety, fear, and despair: “*I am very scared when my parents are away from me… I think they will never come back and I will be left alone in this world… I think all my body parts have been cut off”.* This was common in all age groups. The children’s relationship with one of the parents (usually the father, 71%) is disrupted or severely disrupted: “*My father has never kissed or hugged me… He says he doesn’t love me or my brother”*—a 9-year-old girl and a 10-year-old boy of low SES. However, the lifeline for these children, which encourages them to try to understand themselves, their relationships, and their situation, is a good relationship with the other parent (usually the mother).

### 3.4. Helpless Mentalizing

In this profile (11 children), the child consciously does not attempt to understand the difficult situation. The child is very disappointed and makes no effort to alleviate their suffering. Although the child suffers greatly from the difficult situation, he or she reacts to it with indifference. The child prefers isolation and loneliness instead of engaging in the situation and trying to control it. For example: “*I do not care if my parents are arguing. I do not involve myself in what they are arguing with, it’s not up to me. I go and play internet games on my phone and immerse myself in it”*—a 10-year-old boy of a low SES. The child finds himself/herself extremely helpless and lonely, and although nothing seems important to the child, this is a psychological collapse. The child deliberately makes no effort to understand his/her mental world and that of others, and his/her responses are essentially unemotional: “*It doesn’t matter what I think or how I feel…it doesn’t matter… I don’t care if my parents love me or not”*—a 9-year-old boy of a low SES. The most important feature of this profile is frustration and a kind of obvious indifference to the situation. In the face of a difficult situation, the typical reaction is: “*I do not know how I feel, but I am like a helpless human being with no hope”.* This was common in all age groups in the two groups. Self-injury (such as banging on the head, punching, or even suicidal thoughts) to reduce emotional stress in difficult situations is common in this profile: “*My parents don’t care about me; they are busy with their work… My mom spends her time with her phone instead of her children… It’s like I don’t exist for them… Sometimes I hit my head hard against the wall or hit my nose with my fist… Sometimes people get so tired and upset that they kill themselves, this way all the hardships will end for them”*—an 11-year-old boy of a high SES. They seem to be heartbroken and cut off from the outside world, unable to understand their mental world and that of others, so they deny their intense anger and emotional suffering through indifference. In this profile, although the child tries to hide their inner suffering and painful feelings, in moments of intense emotional arousal, the child unveils himself through self-injury and sometimes aggressive behaviors, revealing suffering. Suicidal thoughts are significant in children of this profile and they are at risk of serious harm to themselves. In this profile, the child’s relationship with both parents is severely disrupted. What bothers the child more than their parents’ behavioral and verbal violence is the persistent pattern of maltreatment, as well as the disregard for the child’s needs and desires, so that the child feels extremely helpless and lonely: “*My parents keep telling me you have to die…my feeling is so painful that I can’t describe it… I tell myself no one loves me so I have to die… When I fight with the children of the family, my parents leave me and favor someone else… I tell them that I am your child, you should support me, not them… I feel very alone and without anyone”*—an 11-year-old boy of a high SES. For the child, the only way to survive in this very painful and unbearable situation is to deny their needs and desires to be understood, loved, and cared for by the parents. By showing indifference to the suffering, the child tries to reduce his/her pain and make the situation bearable for himself/herself: “*I don’t care if they like me anymore… I don’t care about anything anymore… I amuse myself with something so that I don’t think about it”.* This narrative appeared, with minor differences, in all age groups.

The most important difference between passive mentalizing and helpless mentalizing is that children who exhibit passive mentalizing are still hopeful in terms of their ability to understand and control the situation. Passive children struggle in distress to understand them and the child is disturbed and shows many symptoms of anxiety. These efforts are very painful, and the child shows great confusion at being forced to endure painful situations. In contrast, in the case of helpless mentalizing, the child makes no effort to understand and control a difficult situation. The situation is unbearable for the child, and as a result, the child hides a lot of anger and rage behind a mask of indifference.

Although self-injury is common in both the helpless (95%) and passive mentalizing (about 60%) profiles, the causes of self-injury are different. Self-injury in passive mentalizing children is usually not severe and does not cause serious harm, but helpless children are exposed to serious harm to themselves. Almost all children with a helpless mentalizing profile (90%) reported suicidal thoughts. Children with this profile implicitly or explicitly reported that they have no hope of living and do not want to be alive. They expressed they would like to end their lives (90%) because of the maltreatment from their parents and family members.

### 3.5. Self-Focused Mentalizing

With the self-focused profile of mentalizing (seven children), the child’s perception of the situation is impaired due to the child’s difficulty in considering another’s perspective. That is, “the other” has no place in the child’s mental world or this position is very limited. For example, according to the words of one 8-year-old high-SES child: “*When my parents are arguing, I’m afraid the fights will end badly. I’m afraid Dad will no longer pay my Mom to cook for me and they will not talk to me”*. In this profile, the child communicates primarily to satisfy his or her inner needs. The child expects compensation for the things he or she does for others. The child expects all of their needs to be met unequivocally, and if faced with failure or delay, tries to react with stubbornness, disobedience, and verbal and behavioral aggression. In the face of failure, children with this profile of mentalizing show the greatest behavioral response and emotional arousal compared to other profiles: “*My parents were upset* (the child had recognized the parents’ unhappiness and understood that their unhappiness was not caused by the child but by an external factor). *I spoke to them, but they answered me coldly and ignored me … I said to myself, why are they treating me like this… Why is their behavior not loving? … They were always kind… I also got angry with them and stopped listening to them… My mother explained to me that my uncle had surgery on his eyes and they were very upset because of that… then I got upset and felt sorry for them”*—a 9-year-old high-SES child. The children for whom this profile has been identified were divided into two groups. The first group were pampered children who were highly regarded by caregivers and whose needs were met as soon as possible. Their relationships with both parents were good and they showed high self-esteem. The apparent feature in them was their inability to tolerate deprivation and failure. Other people were seen as a means of satisfying their desires. They did not notice their behavioral consequences until they saw the consequences of their behavior obviously and visibly, and empathy was aroused in them only at this time. The other group in the self-focused mentalizing profile (who focused on themselves and their needs in such a way that they could be called narcissists) were children whose relationships with one or both parents were disturbed and whose parent or parents were unresponsive to their child’s needs and punished them. These children had low self-esteem, and in cases of deprivation and failure, they reacted with revenge and verbal and behavioral violence. Also, these children were very competitive and could not stand defeat: “*When I play with other guys, I have to win at all costs, even if I cheat, I have to win… when I’m losing, I start a fight and leave the game because I don’t want to lose”*—a 10-year-old boy and an 11-year-old boy of high SES. In general, in self-focused or narcissistic mentalizing, the child seems to be complex regarding their mental world, with no space to consider other people’s mental states. The child wishes to achieve his/her desires in any way, and in case of failure, the child resorts to aggression, disobedience, and using ineffective strategies to achieve his/her desires.

### 3.6. Differences in Mentalizing Profiles among Typically Developing Children and Clinical Population and Different SESs

To find the dominant sub-theme in each interview, SES was considered an important variable, because research has shown that socioeconomic status has a significant effect on children’s RF [15]. There were also differences in mentalizing profiles between the typically developing children and the clinical population. We reported the frequency and percentage of each profile in the two groups to alert readers to the difference in sample size in the groups and to show the frequency of each profile in each of the two groups. Therefore, we do not intend to compare both groups of the typically developing children and the clinical population, but rather we aim to demonstrate the mentalization profiles of each group independently of each other. See Table 2 to compare the sample sizes and percentages for the two groups. The predominant percentage of adaptive mentalizing in the typically developing children was 67% (76% high SES and 24% low SES), and in the clinical population it was 13%. The predominant percentage of passive mentalizing in the typically developing children was 26% (17% high SES and 83% low SES) and this was 58% in the clinical population. The predominant percentage of helpless mentalizing in the typically developing children was 2% (100% low SES) and this was 29% in the clinical population. The predominant percentage of self-focused mentalizing in the typically developing children was 5% (83% high SES and 17% low SES) and in the clinical population this was 3%. Chi-squared analyses in two adaptive and maladaptive groups indicated that there is a significant difference in the percentage of adaptive or maladaptive metalizing in typically developing high-SES, typically developing low-SES, and clinical children

## 4. Discussion

This study examined how 153 Iranian children aged 8 to 11 from typically developing and clinical populations understand, process, and respond to attachment-related challenges based on CAI interviews. The CAI interviews were transcribed and qualitative content coding was applied, revealing four distinct mentalizing profiles. Differences were also found between the typically developing children and the clinical population, such that the clinical population evidenced more maladaptive RF profiles and the typically developing children showed more adaptive RF profiles. In addition, the children living in low-SES environments displayed more maladaptive mentalizing profiles.

Mentalization in Iran has only recently gained ground, testifying to the novelty and importance of this study’s sample and its results. Given that mentalization is an evolutionary capacity dependent on context, it is necessary to examine RF in different cultural contexts [2]. RF theorists in the context of Western culture have studied mentalization deficits in adults and shown that mentalizing failures are characterized by pre-mentalization, lack of interest and curiosity, and uncertainty [30]. Such theorists have also identified three styles of attachment representation in parent–infant relationships in their studies: hostile, idealizing, and helpless attachment representations. These are incompatible representations that lead to the formation of three types of mentalization deficits in adulthood: hostiles, helpless, and narcissistic. These three states of failure in the mind predict the maladaptive behavior of the parents and the disorganization of the infant. However, mentalization profiles have not been identified and classified for either children or in the context of Eastern culture, and ours is the first study to address this.

It seems that the profiles of mentalization identified in this study on Iranian children are consistent with extant research in the literature. In line with the existing research, adaptive and maladaptive mentalization were identified in these Iranian children, mirroring those seen in other parts of the world. However, although the types of mentalizing might replicate extant research, the content of what is being mentalized and the interpersonal dynamics are indeed unique to Iranian culture, expanding what is known about mentalizing within and across cultures. Adaptive and maladaptive mentalizing profiles are developed based on the communication signals and the amount of care and attention received by the child from parents during their life. Just as living in a supportive environment with responsive and receptive parents sensitive to the needs of the child has an important role in the formation of adaptive mentalizing in children, living in detrimental environments and being with unresponsive and non-supportive parents disrupts the development of RF in children, reflected in the three maladaptive profiles [31]. In helpless mentalizing, the child does not want to lose his/her mental cohesion, and perceives that processing the deplorable situation in which the child finds himself/herself is the only way to survive. So, the dominant RF in this profile is non-mentalizing. In the passive profile, too, the dominant RF is non-mentalizing [32]. Although the child wants to understand and process the situation to realize the reason for his/her confusion and heal his/her mental suffering, the child is not able to do it and is thus trapped in teleological thinking, in which the world is divided into good and bad for the child. The child sees each crisis as catastrophic and his/her interpretations are catastrophic [33]. The child interprets family challenges or any behavior that is not accompanied by love and attention as a reason for misery and rejection by others, as if any challenge or crisis attacks the child’s integrity [34]. Children who experience complex and relationship trauma, especially if the traumatic events begun before their birth, are usually hypervigilant and restless [35]. They have limited space to distinguish between internal and external reality. They have to separate the connection between their internal states and their intolerable external reality because this is the only possible way for them to survive. They have great difficulty in coherently expressing what they are experiencing because they lack words and narratives to help them understand why they are disturbed [36]. The children with a narcissistic profile seem to fluctuate between a psychic equivalence mode and teleological mode because, on the one hand, these children are self-oriented and far from any other’s mental world. The narcissistic profile child does not attempt to test the correctness of thoughts and beliefs or alternative ways, because, for this child, what the child thinks and feels is reality. Competition is very real, even in games, and the child cannot tolerate defeat and wants to win in any way possible [37]. For this child, only the result or achieving what the child wants is important. Others are seen as a means to an end. The child will try to achieve what he desires, regardless of the effect it may have on others [38]. Although the passive and self-focused profiles both represent teleological thinking, each focuses on specific aspects of this thinking. Therefore, RF deficiency poses a major vulnerability in children. Children with maladaptive profiles experience maltreatment. Facing abuse in the complex interaction of environmental, biological, and psychosocial factors reduces the arousal threshold of the attachment system and inactivates controlled mentalization in individuals, and disrupts their ability to differentiate between their mental states and those of others [39,40]. Early biological and neurological stresses disrupt normal brain function and impair key skills such as emotion regulation, impulse control, coping skills, interpersonal skills, and cognition [40].

The findings of this study contribute to the importance of considering mentalization as an important factor in psychopathology or people’s mental health [31]. Understanding the impact of child–caregiver relational patterns and the stressors of life at an early age on the quality of the child’s development and physical and mental health requires a serious focus on early diagnosis and intervention [41]. This study was conducted in a situation where, due to COVID-19, several interviews were conducted over the phone, so it was not possible to interpret the behavior and body language of the child; therefore, only the tone of the child’s voice was analyzed. Another limitation of the study was that only the child was interviewed and no parents were interviewed, so it was not possible to examine the dynamics of the family. Also, we did not examine the effect of the interview format on how the children answered. Finally, inter-rater reliability was not calculated when identifying the mentalizing profiles, but other steps were followed to ensure rigor.

## 5. Conclusions

Mentalization is one of the most important areas of social cognition and plays a very important role in the health or psychological pathology of individuals. In this study, a mixed-method approach was used to examine the ability of children to understand, process, and respond to attachment-arousing situations in typically developing and clinical populations of Iranian children. This study showed that there were differences in understanding, processing, and responding to challenging situations between the two study groups and that the typically developing children evidenced more adaptive profiles of mentalizing, while the clinical population displayed one of three maladaptive profiles of mentalizing. Also, children with a high SES evidenced more adaptive profiles of mentalizing, while children with a low SES evidenced more maladaptive profiles of mentalizing. This study is an important step in increasing our cross-cultural understanding of the development of RF in children and its results can include significant educational and clinical applications. The findings of this study could provide significant implications for child psychotherapists and mentalization-based treatment for children (MBT-C), as they suggest that culture and socioeconomic status are key elements in disorder etiology or treatment outcomes. Also, by considering the role of parents in the formation of adaptive and maladaptive mentalizing profiles, this study highlights the necessity of preventive interventions and training of parents to empower them for effective parenting.

## Figures and Tables

**Table 1:** Attachment-based mentalizing profiles and identified mentalization themes.

Sub-Themes	Main Themes	Master Themes
Maturity and intellectual thinking. “*Sometimes I laugh at my work, now I think about that incident, I laugh, it was a simple incident and I shouldn’t have cried; I think about my behavior to understand what caused this problem; I try to change my wrong behavior”—All age groups.* Role of development in behavioral inhibition and understanding of one’s own and others’ mental states. “*Every child can have different feelings. People’s thoughts are constantly changing. A person should think about good things because thinking about sadness doubles a person’s sadness”—11-year-old boy with a low SES; “When I was little, I couldn’t understand why my mom wouldn’t buy me the things I wanted. I thought she didn’t love me, but now I understand that she just didn’t have money”—11-year-old girl with a high SES; “I take a good revenge on others, a revenge that doesn’t hurt them, only makes them realize their mistake”—10-year-old boy with a low SES.*	Effective processing	Adaptive mentalizing profile
Deliberate efforts to reduce upset with appropriate behavior. “*When my parents argue I try to help them. I tell them funny things/buy them flowers/draw them and try to reconcile them”—All age groups, but especially high-SES children.* Opening her/his heart to another and talking to reduce upset. “*When I am upset or sad, I heartache with my brother/sister/mother/father/friend”—All age groups and both groups.* Pleasant behaviors for emotional management. “*Whenever I don’t have anything I want, I put on makeup. I make up, and my sadness is relieved”—8-year-old girl with a high SES; “I write my sadness to God and calm down”—10-year-old girl.* Thinking of pleasant topics for emotional management. “*When I’m upset, I think of God/my friends/good things to relieve my sadness”—All groups.* Managing emotional states by understanding his/her mental states. “*My brother broke the vase, my mother punished me. I was very sad, I felt that my mother didn’t love me anymore, I felt very lonely, because, it was not my fault. I wanted to hit him but I told myself that I was angry now and if I hit him I might regret it later so I forgave him”—More common in the age groups of 10 and 11 years and high-SES children.*	Impressive coping/controlling strategies	
Understanding the situation. “*My parents argue when they have different tastes. Sometimes they don’t agree and it’s not so important that I want to worry about it; My friend broke my toy, I didn’t say anything, it was just an incident”—In all groups.* Proper understanding of others’ mental worlds. “*She didn’t want to break my doll, it was a simple thing. She was very upset, so I forgave her”—With a little difference in all age groups, especially 10 and 11 years old, and in the two groups.* Understanding other’s worries and mental states. “*I sympathize with my parents… I hide my sadness, I don’t want to upset them”—10-year-old girl; “My parents didn’t talk to me and didn’t answer me… because my mother’s friend had died, they were very upset and sad, that’s why they didn’t answer me”—11-year-old girl with a high SES.*	Efficient understanding	
Internalization of suffering. “*I internalize my sadness and become more and more depressed”—11-year-old girl with a high SES.* Force and desperation to cope with/control a situation. “*When I’m very upset, I don’t show it to myself. I hide my anger. I have to endure the situation/I have to get used to it/I have no choice”—More common in 10- and 11-year-old children.* Fear of not receiving support when needed. “*When I need help, I fear that no one will help me or will not be able to help me; I am afraid to ask others for help and they will say no”—In all age groups, especially younger children and those with a low SES.* Struggle to maintain hope. “*My parents pay more attention to my little brother and I feel very sad that they pay so little attention to me, but I try to keep hope by thinking about my childhood when my parents took care of me all the time and don’t be disappointed”—10-year-old girl with a high SES.*	Scrambling processing	Passive mentalizing profile
Avoiding difficult situations. “*My parents argue, I go and lock myself in my room. I distance myself from my parents… I don’t like to talk or play with anyone”—All age groups, with a slight difference in the narratives.* Difficulty in enduring difficult situations. “*It’s like a part of me is missing. It’s like a part of my body is cut off. Everyone who dies doesn’t come back. How bad it is to die, how bad this world is”—With a slight difference in all age groups.* Struggle to cope with/control a situation. “*I wish I could calm down my parents… God, what can I do to calm down my mother… I tell myself to calm down, calm down, don’t do this ugly thing… I wish I could stop their fight and help them”—All groups.* Denial of a difficult situation. “*I was very saddened by the death of my parrot. For a few days, I amused myself as if it had never existed. I thought my parrot went on a trip and came back”—10-year-old girl with a high SES.* Escape from a difficult situation. “*I run to my room and cover my ears so I don’t hear my parents arguing. I don’t want to think about it. In all groups Struggle to understand your mental state and the other”—Common in all groups.* Wishing for the end of mental suffering. “*I wish I was not in my house. I wish I had a better mom and dad. Happy are those who have a good mom and dad; I wish there was peace at home. I wish my parents would reconcile soon”—All groups.* Difficulty adapting to a difficult situation. “*If my doll is not with me, I will be very lonely as if no one is holding me; I can’t bear to be away from my parents, I cry so much until I pass out; I wither and become sad”—All age groups*.	Passive coping/controlling strategies	
Struggle to understand one’s own mental state and others’. “*I don’t know what I felt like I was somehow, because… I don’t know what I felt. I couldn’t help but cry. I don’t know how I was feeling”—Common in all groups.* Struggle to understand the situation. “*I was confused. Why are they beating me??! I was angry, I couldn’t cry. I couldn’t even know what happened??”*—*10-year-old girl with a low SES.* Difficulty understanding others’ mental states. “*I cannot understand my sister and be kind to her; I don’t know how my mother/father/friend/sister/brother feels when he/she is sad, I can’t understand what she/he is thinking at that moment”—All age groups.*	Disturbed understanding	
Expectation. “*Helping mother is like helping someone who will also try to make better things for me”—11-year-old low SES boy; “I give my things to my brother, I feel he will buy for me many things when he grows up”—9-year-old boy of a low SES.*	Selfish processing	Narcissistic/self-focused mentalizing profile
Psychological suffering in the face of limitations and disabilities. “*I will be very upset if I don’t get what I want. cannot stand it. I hit myself/I get nervous/I insist all the time to get what I want”—All age groups, especially younger children.* Reprisal or stubbornness and disobedience in the face of defeat and failure. “*If I don’t get what I want, I grumble and be stubborn and I don’t listen to them to buy for me”—8-year-old high-SES boy; “If I feel that my parents don’t love me, then I will hurt my sister so that they will pay attention to me”—10-year-old boy with a high SES.*	Hostile coping/controlling strategies	
Understanding the situation based on inner needs and desires. “*My mother gets upset with me, I’m afraid she won’t cook for me”—8-year-old boy with a high SES; “I was upset, the eggs didn’t turn into chicks because I wanted to sell the chicks to get rich”—11-year-old boy with a high SES.*	Self-centered understanding	
Despair and helplessness in enduring situations. “*When my toy breaks, I feel worse and sadder than ever… like a stormy night”—10-year-old boy with a low SES, and, a little differently, an 11-year-old girl with a low SES.* Feeling abandoned and alone. “*I don’t care if they don’t like me, I think no one is more miserable than me; I think no one in the world loves me; I have no feelings except sadness like a cut-down tree”—All groups.* Feeling helpless. “*I can’t say about myself. I have nothing to say about myself. I’m sick, like I’ve been left in a storm”—11-year-old boy with a low SES; “When I’m sad, I feel alone. I like to cry as if there is no one to help me”—11-year-old girl; “I am like a broken vessel that has no hope”—10-year-old boy with a low SES*.	Desperate processing	Helpless mentalizing profile
Self-harm to reduce stress beyond capacity. “*I’m upset I grab my face/I hurt myself/I get very angry and hurt myself. I beat myself/slam my head on the ground, I get dizzy and it makes me even more angry”—All age groups.* Indifference to a difficult situation due to inability to understand the situation. “*I don’t know why my parents are fighting… I don’t care… it’s none of my business. I just watch them fight”—11-year-old boy and 9-year-old girl of low SES.* Isolation. “*I’m sad, I go to my room, I like to be alone, I lock myself in my room”—Common in all age groups.*	Self-destructive coping/controlling strategies	
Despair and helplessness in understanding the situation. “*I don’t know why my parents fight… I don’t want to know why they fight… I don’t care about anything anymore… I don’t even cry anymore”—11-year-old boy of a low SES.*	Blocked understanding	

**Table 2:** Frequency of attachment-based mentalizing profiles and chi-square on the bases of SES and psychopathology.

Profiles	Typically Low SES = 49		Typically High SES = 73		Clinical Population N = 31		Chi-Square			
	N	P	N	P	N	P	RF in Type	Value	Df	Sig
Adaptive	20	40.82%	62	84.93%	4	12.90%	Adaptive	52.80	2	0.000
Passive	26	53.06%	6	8.22%	17	54.84%	Maladaptive			
Helpless	2	4.08%	*	*	9	29.03%				
Self-focused	1	0.02%	5	6.85%	1	3.22%				

*, *n* = 0, which means the frequency is 0.

## Data Availability

The data presented in this study are available on request from the corresponding author. The data are not publicly available due to privacy or ethical restrictions.
